# Association between ranolazine therapy and cognitive decline in elderly patients with ischemic heart disease

**DOI:** 10.3389/fphar.2025.1664988

**Published:** 2025-11-28

**Authors:** Giandomenico Severini, Giuseppe Armentaro, Velia Cassano, Valentino Condoleo, Carlo Alberto Pastura, Alberto Panza, Carlo Fuoco, Sofia Miceli, Vanessa Teresa Fiorentino, Maria Perticone, Raffaele Maio, Elena Succurro, Francesco Andreozzi, Giorgio Sesti, Angela Sciacqua

**Affiliations:** 1 Geriatrics Division, “Renato Dulbecco” University Hospital of Catanzaro, Catanzaro, Italy; 2 Department of Medical and Surgical Sciences, University Magna Græcia of Catanzaro, Catanzaro, Italy; 3 Research Center for the Prevention and Treatment of Metabolic Diseases (CR-METDIS), University Magna Græcia, Catanzaro, Italy; 4 Department of Clinical and Molecular Medicine, University Rome-Sapienza, Rome, Italy

**Keywords:** elderly, ischemic heart disease, cognitive impairment, comprehensive geriatric assessment, ranolazine

## Abstract

**Introduction:**

Ischemic Heart Disease (IHD) represents one of the major causes of mortality and morbidity in the geriatric population This clinical complexity of these patients substantially impacts their quality of life and is associated with an increased risk of cognitive impairment (CoI). Ranolazine, plays a pivotal role in managing anginal symptoms and improving exercise tolerance.

**Objective:**

To investigate the potential protective effect of Ranolazine on CoI over time in elderly patients with IHD and multiple comorbidities.

**Methods:**

We performed a single-center, prospective, observational cohort study. The primary endpoint was a reduction in MMSE score ≥3 points during follow-up compared to baseline values.

**Results:**

519 patients with a mean age of 74.2 ± 6.8 years were enrolled and divided into two groups based on Ranolazine use. The groups demonstrated comparable distribution by sex; however, Ranolazine group, although younger, displayed increased severity of anginal symptoms (Canadian Angina Scale 2.6 vs. 2.3; p < 0.0001), higher prevalence of previous acute coronary syndrome (p < 0.046), sarcopenia (p < 0.0001), and type 2 diabetes mellitus (p < 0.040). During a 4-year follow-up, 186 cases of CoI were observed (8.9 events/100 patient-years) in the general population. The incidence of CoI was significantly lower in the Ranolazine group compared to the control group (5.7 vs. 10.3 events/100 patient-years; p < 0.001). Multivariate analysis revealed a statistically significant association between CoI and the use of Ranolazine, GLP-1RAs, and SGLT2i. Specifically, Ranolazine use was associated with a 61% odds reduction in CoI.

**Conclusion:**

The use of Ranolazine is associated with a significant odds reduction in CoI in elderly patients with IHD and multiple comorbidities. The neuroprotective effect of Ranolazine may be attributed to the improvement of anginal symptoms and consequent optimization of functional status and quality of life, advocating for a comprehensive therapeutic strategy in geriatric patient management.

## 1 Introduction

### 1.1 Global burden of IHD in elderly patients

Ischemic Heart Disease (IHD) is a significant cause of mortality and morbidity. Incidence is expected to increase in the coming years, mainly due to an increase in the elderly population, which will have a negative impact on clinical outcomes and quality of life. A recent study estimated that globally, ischemic heart disease affects around 126 million individuals, with a crude prevalence rate of 1.655/100.000 people and expected to exceed 1.845 by 2030 (Khan et al., 2020).

### 1.2 Association between IHD, comorbidities, and CoI

Aging is a non-modifiable risk factor for cardiovascular disease and has been linked to increased inflammation, known as “inflammaging.” This condition is concomitant with a range of comorbidities, including heart failure, arterial hypertension, diabetes mellitus, chronic kidney disease, and sarcopenia that has been shown, especially in older people, to increase both the risk of IHD and cognitive impairment (CoI) (Liberale et al., 2020; Madhavan et al., 2018; Alonso Salinas et al., 2024).

### 1.3 Current treatment strategies for angina

Managing elderly patients with IHD could be challenging at the same time due to severe comorbidities, often limited treatment options, and atypical symptoms. The treatment for elderly patients with IHD is intended to improve quality of life, life expectancy, and the prevention of cardiovascular events. The initial therapeutic approach, suggested by guidelines, involves the administration of beta and calcium channel blockers as the preferred initial treatment option; Ranolazine should be considered as adjunctive therapy in cases of uncontrolled symptoms or as primary therapy for specific patients (Maron et al., 2020; Vrints et al., 2024; Santucci et al., 2020; Rousan and Thadani, 2019). In particular, Ranolazine, a late Na + current inhibitor, improves cardiac microcirculation and reduces tissue oxygen consumption with no significant effect on hemodynamic parameters and heart rate, reducing angina symptoms and improving exercise tolerance and quality of life (Tamargo and Lopez-Sendon, 2022; Manolis et al., 2024; Mehta et al., 2022; Timmis et al., 2006).

### 1.4 Potential effect of ranolazine on CoI

Particularly in the elderly, another intriguing aspect is the correlation between IHD and cognitive function. Indeed, several studies have demonstrated an association between IHD and an elevated risk of CoI secondary to vascular dementia (Liang et al., 2021). However, to date, we have no evidence on the possible neuroprotective role of antianginal therapy, particularly Ranolazine, on the onset of CoI. Some interesting data come from *in vivo* studies that indicate the neuroprotective effects of Ranolazine, probably due to anti-inflammatory and anti-apoptotic effects on brain tissue (Cassano et al., 2020; Samir et al., 2024).

Nevertheless, the available data on the real benefit of Ranolazine on cognitive function in elderly patients with IHD remain controversial. Considering the aforementioned factors, We hypothesize that Ranolazine use in elderly IHD patients with multiple comorbidities is associated with a slower rate of cognitive decline compared to standard therapy.

## 2 Materials and methods

### 2.1 Study design and patients’ selection

In this prospective observational study, 519 Caucasian patients were enrolled, between December 2017 and August 2023, at the Geriatric Department of “Renato Dulbecco” University-Hospital of Catanzaro. The study included patients suffering from IHD from the Catanzaro Metabolic Risk factors (CATAMERI), a longitudinal observational study assessing cardio-metabolic risk in individuals. The inclusion criteria were age>65 years; written informed consent; history of IHD. Exclusion criteria were: acute coronary syndrome in the previous 3 months, respiratory failure, severe renal dysfunction (estimated glomerular filtrate (eGFR) < 30 mL/min/1.73 m^2^); nephrotic syndrome, macroalbuminuria, severe hepatic impairment (Child–Pugh Class C); pregnancy or breastfeeding, prior diagnosis of dementia or severe psychiatric disorders. Follow-up visits were scheduled every 1 year, with a time window of 4 weeks (±4 weeks); and a pre-defined maximum follow-up of 4 years. A careful medical history was obtained in all subjects. A complete physical examination was performed, and both body weight and body mass index (BMI) were also measured. The study was approved by the local Institutional Ethics Committees, (code protocol number 2012.63). All patients signed informed consent, and the study procedures were carried out in accordance with the principles of the Declaration of Helsinki.

### 2.2 Laboratory parameters

Blood samples were collected after at least 12 h of fasting on peripheral blood samples. Serum Albumin was measured with a colorimetric spectrophotometric method (Bromocresol green). Glycaemia was determined by the glucose oxidase method (glucose analyzer, BeckmanCoulter, Milan) and the homeostasis model assessment (HOMA) index was used for the determination of Insulin resistance (Matthews et al., 1985). Enzymatic methods (Roche Diagnostics GmbH, Mannheim, Germany) were used for determination of blood levels of total cholesterol, low-density lipoprotein (LDL) cholesterol, high-density lipoprotein (HDL) cholesterol, and triglycerides. Alanine aminotransferase (ALT), aspartate aminotransferase (AST) by pyridoxal phosphate activated (liquid reagent), and gamma-glutamyltransferase (γ-GT) were evaluated by standardized method (COBAS Integra 800-Roche Diagnostics GmbH, Mannheim, Germany). Creatinine was measured using the Jaffé method. The CKD-EPI (Chronic Kidney Disease Epidemiology Collaboration) equation was used for the estimation of glomerular filtration rate (eGFR) (Levey et al., 2009). Serum uric acid (UA) levels were assessed using URICASE/POD method (Boehringer Mannheim, Mannheim, Germany). The immuno-turbidimetric method automated system (Cardio Phase hs-CRP, Milan, Italy) was used to assess the high-sensitivity C-reactive protein (hs-CRP).

### 2.3 Primary endpoints

The incidence of CoI during follow-up, defined as reduction ≥3 pt of Mini-Mental State Examination (MMSE) from baseline to follow-up (Dewan et al., 2024), was identified as a study endpoint. Data pertaining to CoI was collected during the follow-up period. In the event of an occurrence, a standardized form was completed by the examining physician. The details of each event were recorded, as well as death certificates, hospital discharge letters or copies of hospitalization medical records, and other clinical documentation obtained from patients or their relatives.

### 2.4 Comprehensive geriatric assessment (CGA)

All patients underwent a CGA. Specifically, cognitive function was assessed using the Mini-Mental State Examination (MMSE) (Folstein et al., 1975), while the presence of depressive symptoms was estimated using the Geriatric Depression Scale (GDS) (Yesavage et al., 1982-1983). Functional status was assessed using activities of daily living (ADL) (Katz et al., 1963) and instrumental activities of daily living (IADL) (Lawton and Brody, 1969). The severity of the angina symptoms was appropriately assessed using the Canadian Angina Scale (CAS) (Campeau, 1976).

### 2.5 Statistical analysis

Data were expressed as mean ± standard deviation (SD), median and interquartile range (IQR), and number and percentage for categorical variables, when appropriate. Student’s t-test was performed for unpaired data for continuous variables, Mann–Whitney’s test for unpaired data for non-continuous variables and χ2 tests for categorical variables. According to Ranolazine therapy, the overall population was divided into two groups. The incidence of Cognitive events was evaluated as the number of events per 100 patient-year. A binary logistic regression analysis was performed on the incidence of CoI evaluated as reduction of MMSE ≥3 pt; subsequently, clinical significant variables and the variables that significantly associated with the occurrence of CoI were included in a multivariate stepwise logistic regression analysis to calculate the odds ratio (OR) for the independent variables associated with the incidence of CoI. Analysis was corrected for age, sex, comorbidities and pharmacological treatments. The differences were considered statistically significant for p value < 0.05. All analyses were performed using the SPSS 20.0 statistical program for Windows (SPSS Inc., Chicago, IL, United States).

## 3 Results

### 3.1 Study population

**FIGURE 1:**
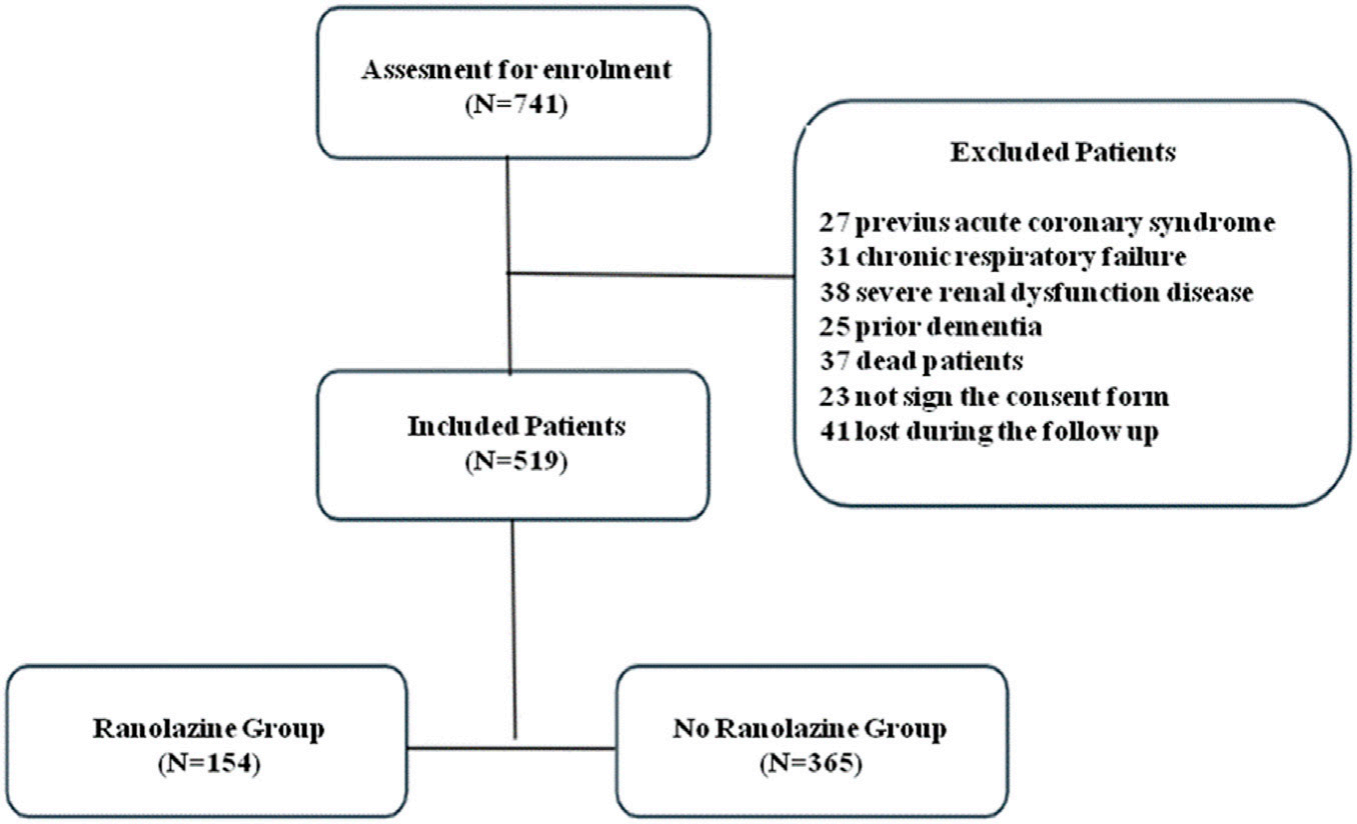
Patients’ selection flowchart.

From an initial population of 741 patients, 27 had acute coronary syndrome in the previous 3 months, 31 had chronic respiratory failure, 38 had severe renal dysfunction disease, and 25 had prior dementia; moreover, 37 confirmed deaths, 23 did not sign the consent form and 41 patients were lost during the follow up; these patients were excluded from analysis (Figure 1). Therefore, 519 patients with an average age of 74.2 ± 6.8 years were enrolled in the study for a follow-up of 4 years. Of these patients, 385 were male, 154 subjects were treated with Ranolazine, and 365 were not receiving this medication (control group). Table 1 shows the comorbidity and medical therapy of the entire study population. Table 2 shows the study population’s clinical, epidemiological, laboratory, and echocardiographic basal parameters, stratified by Ranolazine’s use. Statistically significant differences between the two groups were observed for number of patients with age≥75 years (p < 0.001) more represented in the no-Ranolazine group, prevalence of history of acute coronary syndrome (p < 0.046), sarcopenia (p < 0. 0001) and type 2 diabetes mellitus (T2DM) (p < 0.040) were more prevalent in Ranolazine group (Table 1). The Ranolazine group showed a higher CAS (p < 0.0001) that reflects more severe symptoms in this group compared to the non-Rolazine group.

**TABLE 1:** Comorbidity and medical therapy of study population in relation to Ranolazine intake.

Variables	All population (n. 519)	Ranolazine (n. 154)	No ranolazine (n. 365)	p*
ACS, *n (%)*	265 (51.1)	89 (57.8)	176 (48.2)	**0.046**
ACS-STEMI, *n (%)*	131 (25.2)	44 (28.6)	87 (23.8)	0.257
CABG, *n (%)*	187 (36)	57 (37)	130 (35.6)	0.762
Recurrent SCA/new revascularization, *n (%)*	157 (30.3)	43 (27.9)	114 (31.2)	0.453
Sarcopenia, *n (%)*	116 (22.3)	64 (41.6)	52 (14.2)	**<0.0001**
HF, *n (%)*	279 (53.8)	80 (51.9)	199 (54.5)	0.591
VHD, *n (%)*	128 (24.7)	42 (27.3)	86 (23.6)	0.370
Arterial hypertension, *n (%)*	356 (68.6)	98 (63.6)	258 (70.7)	0.114
PAD, *n (%)*	126 (24.3)	36 (23.4)	90 (24.7)	0.705
AF, *n (%)*	104 (20)	35 (22.7)	69 (18.9)	0.343
Cerebrovascular disease, *n (%)*	143 (27.6)	49 (31.8)	94 (25.8)	0.172
Dyslipidemia, *n (%)*	403 (77.6)	113 (73.4)	290 (79.5)	0.129
SAS, *n (%)*	152 (29.3)	52 (33.8)	100 (27.4)	0.145
COPD, *n (%)*	159 (30.6)	42 (27.3)	117 (32.1)	0.280
T2DM, *n (%)*	298 (57.4)	99 (64.3)	199 (54.5)	**0.040**
ICD-CRT-D, *n (%)*	38 (7.3)	12 (7.8)	26 (7.1)	0.789
ACEi/ARBs, n (%)	375 (72.3)	104 (67.5)	271 (74.2)	0.119
MRA, n (%)	171 (32.9)	66 (42.9)	105 (28.8)	**0.002**
Sacubitril/Valsartan, n (%)	77 (14.8)	27 (17.5)	50 (13.7)	0.262
Statins, n (%)	485 (93.4)	151 (98)	334 (91.5)	**0.006**
PCSK9-i/Inclisiran, n (%)	114 (22)	43 (27.9)	71 (19.5)	**0.033**
Nitrates, n (%)	88 (17)	36 (23.4)	52 (14.2)	**0.011**
β-blockers, n (%)	370 (71.3)	142 (92.2)	228 (62.5)	**<0.0001**
Metformin, n (%)	242 (46.6)	92 (59.7)	150 (41.1)	**<0.0001**
GLP-1RAs, n (%)	108 (20.8)	48 (31.2)	60 (16.4)	**<0.0001**
SGTL2i, n (%)	164 (31.6)	60 (39)	104 (28.5)	**0.019**
Insulin, n (%)	105 (20.2)	31 (20.1)	74 (20.3)	0.970
DPP4, n (%)	110 (21.2)	31 (20.1)	79 (21.6)	0.700
Diuretics, n (%)	272 (52.4)	78 (50.6)	194 (53.1)	0.602
Antiplatelet, n (%)	406 (78.2)	121 (78.6)	285 (78.1)	0.902
OAC, n (%)	119 (22.9)	34 (22.1)	85 (23.3)	0.765

* Performed by chi-square test. Bold numeric characters indicate a statistically significant difference between the two groups.

Abbreviations–ACS-STEMI: acute coronary syndrome - ST-segment elevation myocardial infarction; CABG: coronary artery bypass graft surgery; HF: heart failure; VHD: valvular heart disease; PAD: peripheral artery disease; AF: atrial fibrillation; T2DM: diabetes mellitus type 2; COPD: chronic obstructive pulmonary disease; SAS: sleep apnea syndrome; ICD-CRT-D: implantable cardioverter defibrillators and cardiac resynchronization therapy; ACEi: Angiotensin II, converting enzyme inhibitor; ARBs: Angiotensin II, receptor blockers; MRA: mineralocorticoid receptor antagonist; PCKS9i: Proprotein convertase subtilisin kexin type 9 inhibitors; DPP4: Dipeptidyl peptidase 4 inhibitors; SGLT2i: Sodium-glucose co-transporter-2, inhibitors; GLP-1RAs: Glucagon like peptide 1 receptor agonists; OAC: oral anticoagulant.

**TABLE 2:** Clinical, epidemiological, laboratory and echocardiographic basal parameters of study population in relation to Ranolazine use.

Variables	All population (n. 519)	Ranolazine (n. 365)	No ranolazine (n. 154)	Standardized mean difference (Hedges’g)	p^ǂ^
Male sex, *n (%)*	385 (74.2)	112 (72.7)	273 (74.8)	—	0.623
Age ≥75 years, *n (%)*	240 (46.2)	54 (35.1)	186 (51)	—	**0.001**
Age, *years*	74.2 ± 6.8	72.3 ± 6.4	75.0 ± 6.8	0.41	**<0.0001**
CAS, *points*	2.3 ± 1.2	2.61 ± 1.1	2.19 ± 1.3	0.36	**<0.0001**
BMI, *kg/m* ^*2*^	30.5 ± 5.4	31.2 ± 5.8	30.2 ± 5.2	0.17	**0.038**
SBP, *mmHg*	133.4 ± 14.5	133 ± 13.4	133.5 ± 14.9	0.03	0.740
DBP, *mmHg*	76.1 ± 10.6	76.6 ± 10.5	75.9 ± 9.9	0.06	0.490
PP, *mmHg*	57.2 ± 12.6	56.4 ± 11.6	57.6 ± 13	0.09	0.354
HR, *bpm*	68.6 ± 14	69 ± 13.3	68.4 ± 14.4	0.04	0.659
RR, *breaths/minute*	15.5 ± 2.3	15.7 ± 2.4	15.4 ± 2.21	0.12	0.268
Hb, *g/dl*	13.5 ± 1.7	13.3 ± 1.9	13.6 ± 1.7	0.16	0.072
Hct, *%*	41.5 ± 5.7	41.2 ± 6	41.7 ± 5.5	0.08	0.295
PLT, *10* ^*3*^ */mm* ^*3*^	242.9 ± 85.9	247.7 ± 91.9	240.9 ± 83.2	0.07	0.407
Na, *mmol/l*	137.9 ± 4.35	137.2 ± 4.5	138.2 ± 4.3	0.22	**0.012**
K, *mmol/l*	4.5 ± 0.7	4.5 ± 0.8	4.5 ± 0.7	0.77	0.814
HbA1c, *%*	6.9 ± 1.1	6.9 ± 1.2	6.8 ± 1	0.08	0.475
Creatinine, *mg/dl*	1.1 ± 0.3	1.2 ± 0.2	1.1 ± 0.3	0.42	0.466
eGFR, *ml/min/1.73m* ^*2*^	63 ± 16.8	68.1 ± 22.3	63.7 ± 23.4	0.19	0.125
Albumin, *mg/dl*	4.08 ± 0.6	4.0 ± 0.6	4.1 ± 0.6	0.16	0.284
LDL, *mg/dl*	70.4 ± 14	67.1 ± 14.7	71.8 ± 13.6	0.32	**0.001**
HDL, *mg/dl*	44.5 ± 7.4	43.8 ± 7.5	44.8 ± 7.3	0.13	0.139
Tryglicerides, *mg/dl*	122.8 ± 40.9	128.2 ± 43.1	120.6 ± 39.8	0.18	0.053
NT-pro-BNP, *pg/ml*	187.6 ± 385.2	201 ± 404.1	181.9 ± 352.6	0.04	0.606
Uricemia, *mg/dl*	6.5 ± 1.8	6.5 ± 1.7	6.5 ± 1.9	0.01	0.827
hs-CRP, *mg/l*	3.4 ± 3	3.2 ± 3.1	3.5 ± 2.9	0.09	0.318
LVEF, *%*	48.9 ± 8.7	49.2 ± 8.9	48.2 ± 7.9	0.11	0.227
E/e’	12.5 ± 2.6	13.9 ± 3.7	14.8 ± 4.2	0.23	**0.026**
GLS, *%*	−14.2 ± 3.2	−11.5 ± 1.3	−11.5 ± 1.6	0.01	0.585
GWE, *%*	84.5 ± 6.2	88.4 ± 2.8	88.3 ± 2.8	0.03	0.968
MMSE, *pt*	25.1 ± 1.9	24.9 ± 1.6	25.2 ± 1.9	0.17	0.190
GDS, *pt*	6.2 ± 3.1	6.5 ± 2.5	6.0 ± 3.2	0.18	0.075
IADL, *pt*	6.9 ± 1.1	7.1 ± 1	6.8 ± 1.2	0.28	**0.004**
ADL, *pt*	5.3 ± 0.7	5.4 ± 0.6	5.3 ± 0.7	0.15	**0.036**

* Performed by t-test for unpaired data. Bold numeric characters indicate a statistically significant difference between the two groups.

Abbreviations–CAS: canadian angina scale; BMI: body mass index; SBP: systolic blood pressure; DBP: diastolic blood pressure; HR: heart rate; RR: respiratory rate; Hb: hemoglobin; Hct: hematocrit; PLT: platelet count; Na: sodium; K: potassium; HbA1c: glycated hemoglobin; eGFR: estimated glomerular filtration rate; LDL: low density lipoprotein; HDL: high density lipoprotein; NT-pro-BNP: N-terminal cerebral natriuretic peptide; hs-CRP: high sensitivity c-reactive protein; LVEF: left ventricular ejection fraction; E/A: E wave (peak velocity blood flow from left ventricular relaxation in early diastole) and A wave ratio (peak velocity blood flow from atrial contraction); E/e’: E wave and A wave ratio (parameter for diastolic function assessment); GLS: global longitudinal strain; GWE: global myocardial work efficiency; GDS: geriatric depression scale; IADL: instrumental activities of daily living; ADL: activities of daily living.

### 3.2 Clinical outcomes

**FIGURE 2:**
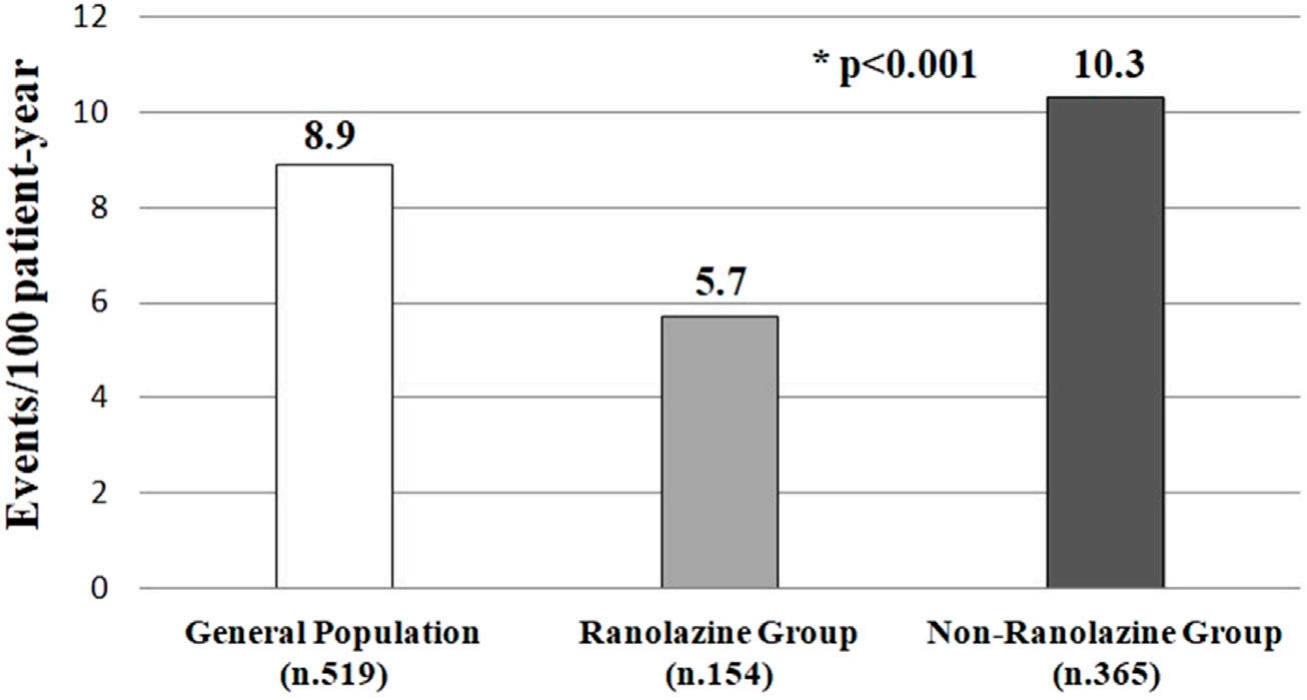
Incidence of CoI after 4 years’ follow-up. Abbreviation: CoI, Cognitive Impairment.

During a follow-up of 4 years, we observed a total of 186 cases of CoI (8.9 events/100 patient-year) in the whole study population. The incidence of the primary outcome was significantly lower in the Ranolazine group versus the control group (p < 0.0001), also considering the incidence rate (35 cases, 5.7 events/100 patient-year vs. 151 cases, 10.3 events/100 patient-year) (p < 0.001) (Figure 2).

### 3.3 Regression analysis

Binary logistic regression analysis revealed a statistically significant association between CoI occurrence and treatment with Ranolazine, GLP-1RAs, and SGLT2i, in addition to the reduction of MMSE, GDS, and Hb values, and the increase of ADL, IADL, and CAS values were significantly correlated (Supplementary Table S1). Consequently, the variables significantly correlated with the dependent variable were entered into a multivariate stepwise logistic regression model to determine the independent predictors of CoI (Table 3). Ranolazine (OR 0.39; CI 0.247–0.640) was particularly interesting, as it was associated with a 61% odds reduction of CoI occurrence. In addition, the following factors were found to be associated with an odds reduction of CoI: 60% for GLP1-RAs use (OR 0.40; CI 0.25–0.65), 57% for 1 point increase in ADL (OR 0.40; CI 0.39–0.73), 40% for SLGT2i utilization (OR 0.60; CI 0.38–0.952), 20% for an increase of 1 point in IADL (OR 0.80; CI 0.67–0.97), 15% for 1 point increase in Hb levels (OR 0.85; CI0.76–0.96) and 11% for 1 point reduction in GDS (OR 0.89; CI 0.83–0.96), respectively. Finally, a 1-point reduction in MMSE (OR 1.26; CI 1.12–1.42) and a 1-point increase in CAS (OR 1.25; CI 1.05–1.48) were associated with an increased risk of CoI for a 26% and 25%, respectively.

**TABLE 3:** Multivariate stepwise logistic regression analysis about incidence of cognitive impairment as dependent variable.

Variables	OR	CI 95%	P
Ranolazine, *yes/no*	0.398	0.247–0.640	<0.0001
GLP-1 RAs, *yes/no*	0.405	0.255–0.656	<0.0001
ADL, *1 pt increase*	0.538	0.393–0.736	<0.0001
SGLT2i, *yes/no*	0.606	0.385–0.952	0.030
IADL, *1 pt increase*	0.809	0.670–0.977	0.028
Hb, *1 gr/dl increase*	0.858	0.764–0.964	0.010
GDS, 1 pt *decrease*	0.896	0.836–0.961	0.002
MMSE, *1 pt decrease*	1.264	1.126–1.420	<0.0001
CAS, *1 pt increase*	1.251	1.058–1.480	0.009

Abbreviations: GLP1-RAs, Glucagon-like-peptide 1 receptor agonists; ADL: activities of daily living; SGLT2i: Sodium-glucose co-transporter-2 (SGLT-2) inhibitors; IADL: instrumental activities of daily living; Hb; Hemoglobin; GDS: geriatric depression scale; MMSE: Mini-Mental state examination; CAS: canadian angina scale.

## 4 Discussion

This study demonstrated that, in elderly patients with IHD and several comorbidities, treatment with Ranolazine was associated with a significant odds reduction of CoI, representing the primary protective factor justifying 61% of odds reduction, even after adjustment for other confounding factors. Of interest, not only use of GLP-1RAs and SGLT2i was associated with a odds reduction of CoI, but also the improvement in functional and humoral clinical conditions represented by CGA tests, at least in part due to Ranolazine treatment was significantly associated with CoI odds reduction.

### 4.1 Neuroprotective effect of ranolazine: what may be the mechanism?

This is the first clinical evidence in humans; data concerning the neuroprotective effect of Ranolazine and the impact of the medication on CoI is primarily drawn from studies conducted *in vitro* or in murine models. The possible neuroprotective effects of Ranolazine in humans may be explained, as demonstrated *in vitro*, by its action on various isoforms of voltage-gated sodium channels, including neuronal isoforms (Theile and Cummins, 2011). Research on primary astrocyte cultures has shown that Ranolazine reduces TNF-α and IL-1β levels and increases PPAR-γ levels, promoting anti-inflammatory and neuroprotective effects by blocking sodium channels at therapeutic doses (Aldasoro et al., 2016). Pro-inflammatory cytokines released by astrocytes have been implicated in the pathogenesis of neurodegenerative diseases such as Alzheimer’s dementia (Fuller et al., 2010). Therefore, Ranolazine may have a neuroprotective role by acting on CoI and dementia. In addition, the transcription factor PPAR-γ, which regulates cellular energy metabolism and inflammation, has been shown to provide neuroprotection in neurodegenerative diseases such as stroke and Alzheimer’s disease (Chen et al., 2012). Even in animal models, Ranolazine has been shown to mitigate not only the cardiotoxicity but also the neurotoxicity induced by doxorubicin. In addition, the neuroprotective effect of Ranolazine has been demonstrated by the reduction of NFkB phosphorylation in the hippocampus, leading to a decrease in inflammation and the reduction of ROS levels with the improvement of mitochondrial dysfunction. In the same study, doxorubicin-induced hippocampal plasticity and cognitive dysfunction were evaluated, and the efficacy of Ranolazine in ameliorating these effects was demonstrated through its actions on dendritic fiber density and synaptophysin levels (Chunchai et al., 2022). Another interesting effect of Ranolazine has been observed on CoI in hyperglycemic rat models, which showed reduced hippocampal neuron degeneration after treatment (Cassano et al., 2022).

It is known that CoI in patients with T2DM has been associated with microvascular dysfunction that results in global atrophy and lacunar infarctions, particularly in cases of long-term diabetes. Notably, hyperglycemia damages the brain’s microvasculature, leading to neuron and myelin loss, reduced endothelial integrity, and astrocyte impairment, all contributing to CoI (Al Hamed and Elewa, 2020; Yan et al., 2020). Moreover, insulin resistance, associated with a chronic inflammatory state mediated by cytokines such as IL1-beta and IL6, is linked to Alzheimer’s dementia and CoI (Kamal et al., 2014).

### 4.2 GLP1-RAs and SGLT2-i: potential protective effects on CoI incidence

The present study further underscores the association between the anti-diabetic medications GLP1-RAs and SGLT2-i and a reduced incidence of CoI. GLP-1 receptor agonists have been demonstrated to reduce the incidence of CoI by acting on the central resistance insulin mechanism. Moreover, these medications have been observed to cross the blood-brain barrier, acting directly on brain regions such as the hippocampus, frontal cortex, thalamus, and hypothalamus (Bassil et al., 2014). Furthermore, studies have shown that these medications can improve neuroinflammation, oxidative stress, and cellular survival (Cheng et al., 2022).

Regarding SGLT2i, they may exert their neuroprotective effects through multiple mechanisms, predominantly involving the cardiorenal axis and ati-inflammatory pathways in elderly patients (Shah et al., 2025). In addition, some authors have proposed a pleiotropic effect, suggesting benefits that extend to the neuroprotective role (Pawlos et al., 2021). In particular, the studies conducted on the MAGIC-HF (MAgna GraecIa Comorbidities in Heart Failure) registry have considered a population of elderly patients with T2DM and heart failure across the whole spectrum of ejection fraction with an IHD prevalence of approximately 60%. They have demonstrated that using SGLT2i improves the different items of CGA (Magurno et al., 2024; Mei et al., 2024). Moreover, evidence suggests that SGLT2i can improve cognitive function and reduce the incidence and progression of mild CoI and dementia in diabetic patients (Lard et al., 2024; Youssef et al., 2023). It has been hypothesized that SGLT2i exert a direct neuroprotective effect through the inhibition of acetylcholinesterase, the increase of brain-derived neurotrophic factor (BDNF) levels, the reduction of oxidative stress and the reduction of the accumulation of amyloid beta plaques, in addition, SGLT2i may improve cerebral insulin sensitivity similar to GLP1-Ras resulting in increased hippocampal synaptic plasticity (Mancinetti et al., 2023; Armentaro et al., 2024). However, further research is required to confirm these actions.

### 4.3 Potential role of ranolazine on symptoms control and functional status in elderly patients

Concerning the impact of IHD symptoms, multivariate analysis indicates a correlation between elevated CAS scores and an augmented odds of CoI. As demonstrated by various studies, preserving optimal physical performance is associated with a reduced odds of CoI and the development of dementia, a crucial consideration for elderly patients (Boa Sorte Silva et al., 2024). The clinical efficacy of Ranolazine in reducing the severity and frequency of IHD symptoms has been demonstrated in several studies, including MERLIN TIMI-36, CARISA, OSCAR, ARETHA, and RANGER (Morrow et al., 2009; Sendón et al., 2012; Alexopoulos et al., 2016; Zweiker et al., 2019; Olympios et al., 2024). These findings align with the results of the present study, which also showed that worsening of angina symptoms, as indicated by a 1-point increase in the CAS score, increases the odds of developing CoI by 25%. In association with tailored exercise, this approach has been shown to enhance physical performance, with a significant reduction in frequency and burden of symptoms, thereby improving the quality of life of IHD patients (Willis et al., 2019). The present study also indicates that a one-point increase in IADL and ADL and a one-point decrease in GDS are associated with a reduced odds of CoI. This finding aligns with the results of the ACTIVE study, which emphasizes the role of self-reported impairments in IADL in predicting more severe CoI and the development of mild CoI and dementia (Tomaszewski et al., 2018).

### 4.4 Future prospective

These observations suggest that the alleviation of angina and IHD symptoms with drugs of proven effectiveness, such as Ranolazine, and the enhancement of physical performance may empower these patients to maintain their autonomy in daily life, thereby mitigating the risk of social isolation and depression, and consequently of CoI occurrence. Although it is important to acknowledge that the primary observations supporting the neuroprotective effect of ranolazine are mainly derived from murine and *in vitro* studies, our results derived from real-world practice with the aim of an integrated management of both cardiovascular and cognitive comorbidities. These representing a promising direction for future healthcare advancements, exploring the potential role of ranolazine and other antianginal drugs not only in reducing the burden of angina symptoms but also the incidence of CoI in elderly patients with IHD, condition which dramatically compromises the quality of life and prognosis of elderly patients.

## 5 Conclusion

This prospective observational study demonstrates that Ranolazine significantly reduced the odds of CoI in elderly patients with IHD. The effect of Ranolazine on CoI can be attributed to its direct action of neuroprotection, particularly in the hippocampus and glial cells, resulting in anti-inflammatory and antioxidant effects on neurons. However, the improvement of angina symptoms and consequently of quality of life, functional independence, and emotional state, at least in part due to Ranolazine, can justify the odds reduction of CoI with Ranolazine.

Nevertheless, it is imperative to recognise the limitations of our study. The present study is prospective, observational and monocentric, a factor which may limit the generalisability of the results. Moreover, there is an absence of data concerning habits and lifestyle, including physical activity, sleep duration and quality, eating habits, alcohol consumption and smoking, recreational activity, as well as information on social activity and familiar networks and stress management. The collection of this information could contribute significantly to a more precise definition of the risk of developing CoI in elderly patients with IHD.

## Data Availability

The raw data supporting the conclusions of this article will be made available by the authors, without undue reservation.
