# Supplementary material for: Association between ranolazine therapy and cognitive decline in elderly patients with ischemic heart disease
Source: Front Pharmacol. 2025 Nov 28;16:1664988. doi: 10.3389/fphar.2025.1664988 (PMC12698426; doi:10.3389/fphar.2025.1664988)
Supplement: Supplementary file 1 [file Table1.docx]

Supplementary Material

**Supplementary Table 1. Linear logistic regression analysis about incidence of Cognitive Impairment as a dependent variable**

|  | **OR** | **CI 95%** | **P** |
| --- | --- | --- | --- |
| **Male,** *yes/no* | 1.194 | 0.736 – 1.938 | 0.473 |
| **Ranolazine,** *yes/no* | 0.349 | 0.205 – 0.593 | **<0.0001** |
| **Age ≥ 75 years,** *yes/no* | 0.820 | 0.539 – 1.247 | 0.354 |
| **Previous ACS,** *yes/no* | 0.755 | 0.492 – 1.160 | 0.200 |
| **MMSE,** *1* *pt decrease* | 1.262 | 1.117 – 1.425 | **<0.0001** |
| **Sarcopenia,** *yes/no* | 1.437 | 0.848 – 2.433 | 0.178 |
| **CAS,** *1 pt increase* | 1.217 | 1.016 – 1.458 | **0.033** |
| **BMI,** *1 Kg/m^2^  increase* | 0.978 | 0.941 – 1.018 | 0.277 |
| **Hb,** *1 g/dl decrease* | 0.866 | 0.768 – 0.977 | **0.019** |
| **LDL,** *1* *mg/dl* | 1.013 | 0.998 – 1.029 | 0.089 |
| **HbA1c,** *1% increase* | 1.018 | 0.785 – 1.319 | 0.893 |
| **E/e’,** *1 pt increase* | 1.040 | 0.960 – 1.125 | 0.338 |
| **MRA,** *yes/no* | 0.835 | 0.520 – 1.343 | 0.458 |
| **Statins,** *yes/no* | 0.795 | 0.328 – 1.926 | 0.611 |
| **PCSK9i/Inclisiran,** *yes/no* | 0.808 | 0.483 – 1.353 | 0.417 |
| **Nitrati,** *yes/no* | 0.711 | 0.412 – 1.228 | 0.221 |
| **β-blockers,** *yes/no* | 0.893 | 0.547 – 1.457 | 0.651 |
| **Metformin,** *yes/no* | 0.932 | 0.555 – 1.565 | 0.791 |
| **GLP-1RAs,** *yes/no* | 0.357 | 0.207 – 0.602 | **<0.0001** |
| **SGLT2i,** *yes/no* | 0.590 | 0.364 – 0.956 | **0.032** |
| **GDS,** *1 pt decrease* | 0.896 | 0.832 – 0.964 | **0.003** |
| **IADL,** *1 pt increase* | 0.806 | 0.662 – 0.981 | **0.032** |
| **ADL,** *1 pt increase* | 0.541 | 0.392 – 0.748 | **<0.0001** |

**Abbreviations – ACS:** acute coronary syndrome; **MMSE:** Mini-Mental state examination; **CAS:** Canadian angina scale; **BMI:** Body mass index; **Hb;** Hemoglobin; **LDL:** low density lipoproteins**; HbA1c:** glycated hemoglobin; **E/e’:** E-wave to e′-wave ratio (reliable estimate of end-diastolic pressure changes); **MRA:** mineralocorticoid receptor antagonists; **PCSK9i:** Proprotein convertase subtilisin kexin type 9 inhibitors; **GLP1-RAs** Glucagon-like-peptide 1 receptor agonists; **SGLT2i:** Sodium-glucose co-transporter-2 (SGLT-2) inhibitors; **GDS**: geriatric depression scale; **IADL**: instrumental activities of daily living; **ADL**: activities of daily living.
